# DTome: a web-based tool for drug-target interactome construction

**DOI:** 10.1186/1471-2105-13-S9-S7

**Published:** 2012-06-11

**Authors:** Jingchun Sun, Yonghui Wu, Hua Xu, Zhongming Zhao

**Affiliations:** 1 Department of Biomedical Informatics, Vanderbilt University School of Medicine, Nashville, TN 37232, USA; 2 Department of Psychiatry, Vanderbilt University School of Medicine, Nashville, TN 37232, USA; 3 Department of Cancer Biology, Vanderbilt University School of Medicine, Nashville, TN 37232, USA; 4 Center for Quantitative Sciences, Vanderbilt University Medical Center, Nashville, TN 37232, USA

## Abstract

**Background:**

Understanding drug bioactivities is crucial for early-stage drug discovery, toxicology studies and clinical trials. Network pharmacology is a promising approach to better understand the molecular mechanisms of drug bioactivities. With a dramatic increase of rich data sources that document drugs' structural, chemical, and biological activities, it is necessary to develop an automated tool to construct a drug-target network for candidate drugs, thus facilitating the drug discovery process.

**Results:**

We designed a computational workflow to construct drug-target networks from different knowledge bases including DrugBank, PharmGKB, and the PINA database. To automatically implement the workflow, we created a web-based tool called DTome (Drug-Target interactome tool), which is comprised of a database schema and a user-friendly web interface. The DTome tool utilizes web-based queries to search candidate drugs and then construct a DTome network by extracting and integrating four types of interactions. The four types are adverse drug interactions, drug-target interactions, drug-gene associations, and target-/gene-protein interactions. Additionally, we provided a detailed network analysis and visualization process to illustrate how to analyze and interpret the DTome network. The DTome tool is publicly available at http://bioinfo.mc.vanderbilt.edu/DTome.

**Conclusions:**

As demonstrated with the antipsychotic drug clozapine, the DTome tool was effective and promising for the investigation of relationships among drugs, adverse interaction drugs, drug primary targets, drug-associated genes, and proteins directly interacting with targets or genes. The resultant DTome network provides researchers with direct insights into their interest drug(s), such as the molecular mechanisms of drug actions. We believe such a tool can facilitate identification of drug targets and drug adverse interactions.

## Background

Currently, the discovery of novel drug candidates is faced with several serious problems, such as a decreased success rate [1] and an increase of the time and expense required [2]. Most often, a limited understanding of the underlying biological mechanisms that cause lower efficacy or adverse side effects leads to these drug discovery issues. Drug efficacy can be affected by the complexity of biological networks, of which targets are only a part; whereas adverse side effects of a drug may be caused by unwanted cross-reactivity with other biologically relevant targets [3,4]. To address these issues, it is vital to obtain a thorough understanding of biological networks, disease-related pathways, and drug-altered complex cellular processes in patients.

Network-based approaches have proved to be one effective means of organizing high-dimensional biology datasets and extract meaningful information [5,6]. Given the complex multivariate processes and advances in pharmacogenomic research, a theoretical foundation for network pharmacology has been proposed [7] and successfully applied to the field of pharmacology [8]. Network pharmacology is defined as a network-centric view of drug actions by mapping drug-target networks onto biological networks, which provides new insights into the role of polypharmacology in drug actions [9]. Network-based approaches have been successfully applied to numerous areas in pharmacology, including novel target prediction for known drugs [10-12], identification of drug repositioning and combination [13-15], and inference of potential drug-disease associations [16]. As these network-based approaches become more and more effective, it is necessary to develop an automated tool to integrate drugs with biological molecules in a network context.

This paper presents a web-based tool that automatically constructs a DTome network for a given drug or set of drugs in order to further explore the molecular mechanisms of drug actions. Considering that protein-protein interactions (PPIs) contain information of the inherent combinatorial complexity of cellular systems, we overlaid the drug targets and drug-associated genes into human PPIs to recruit their directly interacting proteins as potential off-targets. This tool integrated drugs, drug primary targets, drug-associated genes, and target/gene functional associated proteins into a network. We demonstrated the utility of the tool by constructing a DTome network for drug clozapine. To the best of our knowledge, this is the first computational workflow to integrate drug information with PPIs, which may facilitate a better understanding of the molecular mechanisms of drug actions for the identification of new drug targets and the prediction of effective drug combinations and drug adverse events.

## Materials and methods

### Dataset preparation

In this study, a DTome network was designed to include three types of nodes and four types of relationships. The three types of nodes referred to drugs, proteins and genes. Drugs included the candidate drugs and other drugs having adverse interactions with those candidate drugs. The proteins included drug primary protein targets and other proteins that interact directly with targets/genes. The drug primary targets were extracted from DrugBank database [17-19]. Other proteins that interact directly with targets/genes were extracted from human PPI data from the PINA (Protein Interaction Network Analysis) database [20]. The drug-associated genes referred to genes with known pharmacokinetic (PK) and pharmacodynamic (PD) evidence extracted from PharmGKB (The Pharmacogenomics Knowledge Base) database [21]. The four types of relationships included drug-drug interactions, drug-target interactions, drug-gene associations, and target-/gene-protein interactions. The drug-drug interactions were directly compiled from the field of "Drug Interactions" in DrugBank, which indicated that two drugs are known to interact, interfere or cause adverse reactions when they are arranged together. An interaction between a given drug and one of its primary targets was assigned. Similarly, an association between a given drug and one of its associated genes was defined based on the evidence extracted from PharmGKB. The interactions between a target/gene and other proteins were retrieved from human PPI data.

As above mentioned, we mainly utilized data from three databases: DrugBank, PharmGKB, and PINA. DrugBank is a freely available online database that combines detailed drug data with comprehensive drug-target and drug-action information. We utilized DrugBank XML file (version 3.0) downloaded on June 2011 from the DrugBank website [22]. For each drug, we extracted "Drug Interaction" and "Target" data to obtain adverse drug interactions and drug primary targets. In this study, we used the DrugBank drug IDs and drug names to represent drugs and the unique UniProtKB accession numbers (ACs) to represent protein targets.

PharmGKB is another knowledge base database that captures the information about drugs, diseases/phenotypes and genes involved in PK and PD. From this database, we extracted the genes with known PK/PD evidence, which were defined as drug-associated genes. To map these drug-associated genes to drugs from DrugBank, we first directly utilized the Drug External Links files from DrugBank to map PharmGKB drugs. Then, we transferred the unmatched drug names in the DrugBank or PharmGKB into drug generic names using MedEx, an automated medication extraction system for drugs [23], and then manually checked them.

The third database we used, PINA, is an integrated platform of PPI data extracted from six public databases: IntAct [24], MINT [25], BioGRID [26], DIP [27], HPRD [28] and MIPS/MPact [29]. PINA includes self-interactions, interactions predicted by computational methods, and interactions between human proteins and proteins from other species. For the purpose of this study, we first downloaded data from the PINA website (June, 2011) and then filtered the data by requiring PPIs to have experimental evidence, removing redundancy and self-interactions as well as interactions involving proteins from other species. This dataset and its process have been found useful in our many network-based projects [30,31].

To clarify and create consistency among the downloaded datasets, we used Entrez gene symbols to represent genes and proteins. The UniProtKB ACs were transferred to gene symbols via two steps: 1) mapping UniProtKB ACs to Entrez gene IDs by an ID Mapping tool in UniProt database [32]; 2) mapping gene IDs to gene symbols according to the annotation file downloaded from the NCBI human reference genome Entrez Gene [33].

### Database design and implementation

We extracted drug information from the above three databases and organized all the data into an open source MySQL database management system to facilitate a cross-database search. Each data set was saved in the MySQL database as tables that store specific information, whereas primary keys (e.g., DrugBank ID, GeneBank ID and PharmGKB ID) were used extensively for relational links. The online interface was implemented in PHP and JavaScript, and hosted on a Linux Apache web server.

### Network generation and analysis

Through its search function, the DTome tool utilizes user-specified keywords to provide a candidate drug or a list of drugs and generate four types of relationships. Then, it merges these relationships to form a DTome network, which could be further analyzed and visualized using the Cytoscape software (version 2.8.0) [34] or other network analysis tools.

To analyze a DTome network, in the example of clozapine, we integrated multiple network characteristics to identify critical targets and drug-bioactive modules. Those network characteristics included degree, degree distribution, hub, and network module. The degree of a node is the most elementary characteristic in a network, which is measured by the number of links of the node. If the degree distribution of one network follows a power law, the network would have only a small portion of nodes with a large number of links (i.e., hubs) [35]. Hubs in the biological network are more likely to be essential genes, which play important roles in maintaining the overall connectivity of the network [36,37]. To determine the hubs in the network, we first calculated the degree for each node in the DTome network and then plotted the degree distribution of all nodes. Based on the degree distribution, we determined the point where the distribution began to plateau. The nodes with a degree higher than the point are hubs that include drugs and targets. For network module analyses, we grouped the involved proteins into four classes according to clozapine-specific network topology. For the complex drug-target network, we recommend performing cluster analysis by applying the software cFinder, which can find and visualize overlapping dense groups of nodes in a network [38].

### Drug classification and gene set enrichment analysis

To examine the classification characteristics of drugs involved in the DTome network, we grouped them using the Anatomical Therapeutic Chemical (ATC) classification system [39]. The ATC system is used for the drug classification, which is controlled by the WHO Collaborating Centre for Drug Statistics Methodology. The system divides active drugs into five different levels according to the organ or system on which they act and/or their therapeutic and chemical characteristics. The first level of the ATC code has fourteen main groups, i.e. the anatomical main groups. And each group is represented by one letter. For example, N represents nervous system. In the case of clozapine, we utilized the third level of the code, which indicates the therapeutic/pharmacological subgroup.

To assess if proteins involved in the DTome network have functional features, we performed the KEGG (Kyoto Encyclopedia of Genes and Genomes) pathway enrichment analysis implemented in WebGestalt (WEB-based GEne SeT AnaLysis Toolkit) [40]. We selected pathways with an adjusted *P*-value less than 0.01, calculated first using the hypergeometric test and followed by the Benjamini-Hochberg method [41].

## Results

### Overview of the DTome tool

**Figure 1:**
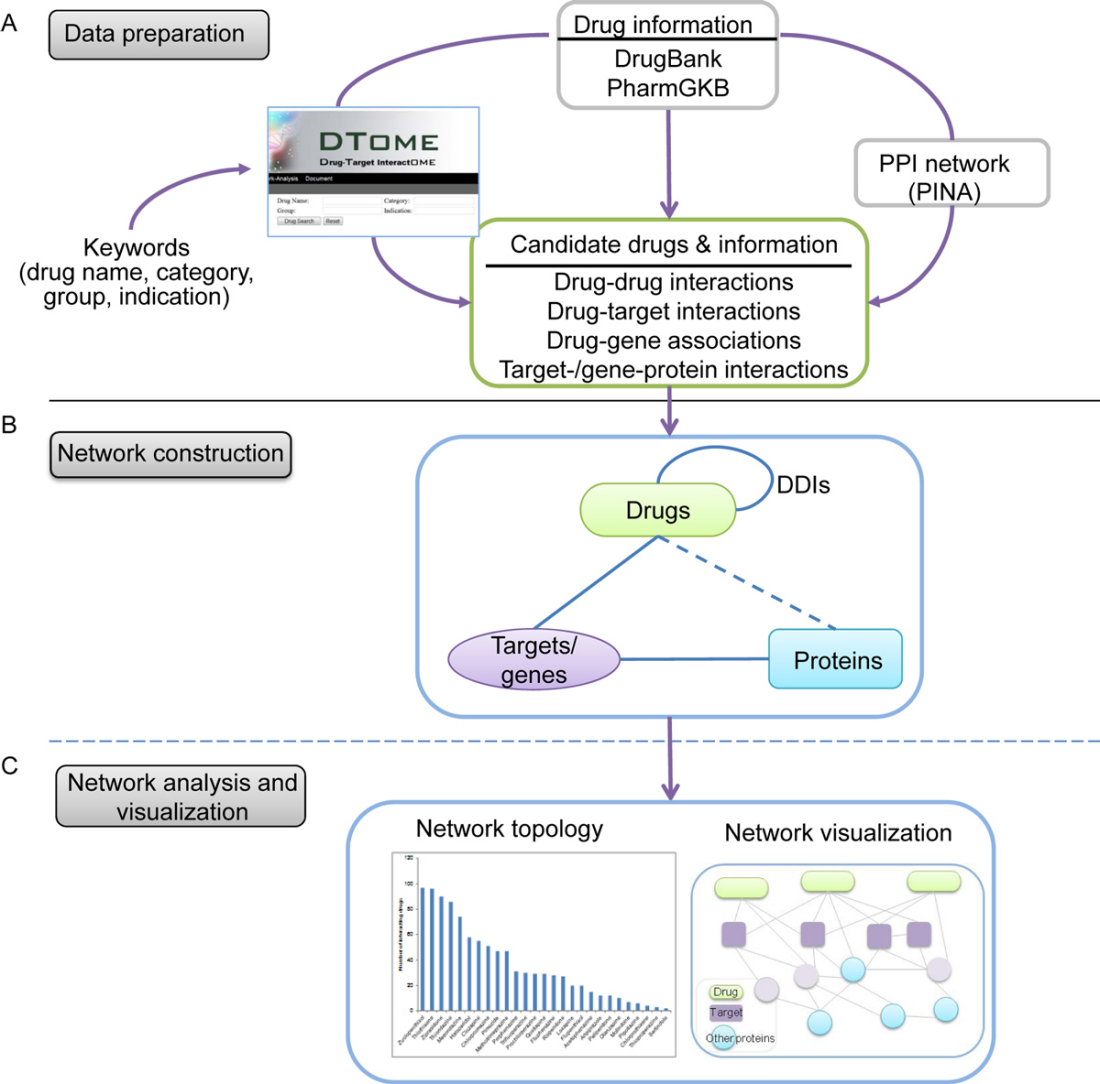
**Overview of DTome: a web-based tool for drug-target interactome construction and analysis**. The DTome network is designed to include three types of nodes (drug, protein and gene) and four types of relationship (adverse drug interaction, drug-target interaction, drug-gene association and target-/gene-protein interaction). The workflow includes three main steps. **A) **Data preparation and database construction. This step includes parsing the data from multiple databases and creation of a database. **B) **Generation of user-specified data and network. The user-specified data include a candidate drug or a list of drugs and four types of interactions. After merging the interactions, a DTome network is formed. **C) **Network analysis and visualization by the Cytoscape software. PINA: Protein Interaction Network Analysis.

As illustrated in Figure 1, the DTome tool provides a computational workflow to integrate candidate drugs with their adverse drug interactions, primary targets, and associated genes in the context of human PPIs. The workflow includes three main steps: dataset preparation and database construction, generation of user-specified data and network, and network analysis and visualization.

**Figure 2:**
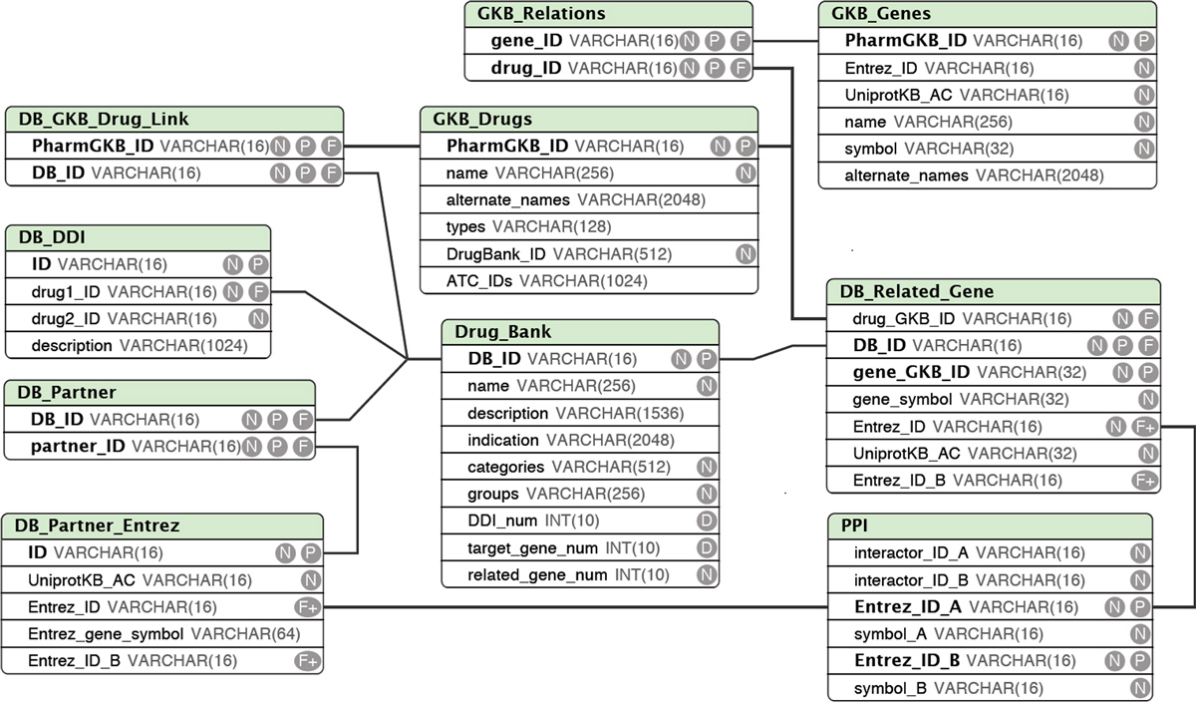
**Database schema**. DB: DrugBank. GKB: PharmGKB. DDI: drug-drug interaction. AC: UniProtKB accession number. ATC: Anatomical Therapeutic Chemical classification system. P: primary key. N: not_NULL. F: foreign key. VARCHAR denotes "variable-length string" type in MySQL. The number in brackets denotes the maximum length of this field.

The first step focused on dataset preparation and database construction based on three databases (DrugBank, PharmGKB, and PINA). Figure 2 shows the detailed database design. The database included 6,796 drugs with unique DrugBank IDs and drug names, 3,848 unique primary targets with gene symbols, 10,931 unique adverse drug interactions, and 73,194 PPIs among 11,656 proteins with experimental evidence. From the Drug External Link files downloaded from DrugBank, 1,135 DrugBank drug IDs were matched with PharmGKB drug IDs. We further matched 433 drugs by transferring the drug names from the DrugBank and PharmGKB to generic names using the MedEx system. Thus, a total of 1,568 drugs were mapped to each other.

After the creation of the database, a candidate drug or a list of candidate drugs could be searched within the database through four options of the individual or joint inquires. The four options are "Drug Name", "Category", "Group", and "Indication", which were adopted from DrugBank. "Drug Name" is the standard name of a drug as provided by the drug manufacturer. "Category" is the therapeutic or general category of a drug, such as anticonvulsant, antibacterial, and so on. "Group" indicates a drug's status, which can be one or more status of the following: "Approved", "Experimental", "Nutraceutical", "Illicit", and/or "Withdrawn". "Indication" is the drug-associated disease. The DTome tool provides drug detail information in the above options for further examination to determine if they are truly candidate drugs. This step is important to determine interactions, follow-up data integration, and further analyses.

From the candidate drug(s), the DTome tool provides an engine to extract four relationships between candidate drug(s) and related molecules mentioned previously (see Materials and Methods). Then, the DTome tool integrates these relationships to form a DTome network and stores it in a text file, which can be downloaded for further network analysis and visualization.

### Web interface

**Figure 3:**
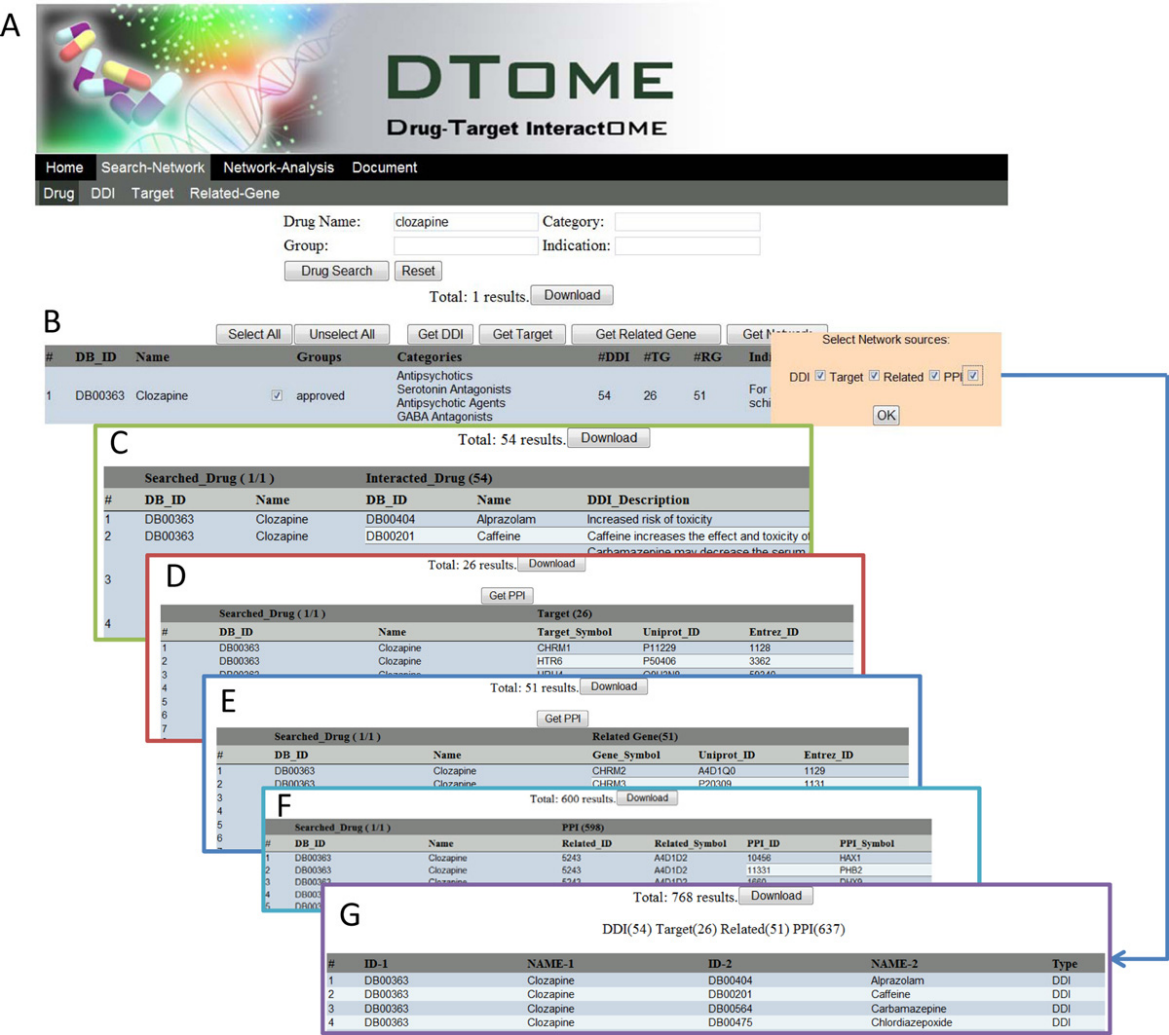
**DTome web interface**. **A) **Drug search page. **B) **Drug search output. **C) **Drug-drug interaction (DDI) output. **D) **Drug-target output. **E) **Drug-associated gene output. **F) **Target-protein output. **G) **DTome network output.

We developed a user-friendly web interface for the DTome tool, which allows users to refine searches based on four options individually and jointly (Figure 3A). In the "Drug Name" option, users can obtain a candidate drug using a whole-word search or a list of potential drugs using a partial-word search. In the "Category" option, users can obtain a list of drugs using a keyword of a therapeutic or general category such as "antipsychotic", "anticonvulsant", or "antibacterial". In the "Group" option, users can obtain a list of drugs using a keyword for drug approval status mentioned previously. In the "Indication" option, users can input a disease name and then obtain a list of drugs that might be used to treat the disease. Additionally, the interface also provides a combinatorial search of above four options. After a keyword search, the output page provides the number of drugs matching users' requirements and a summary table (Figure 3B). For each drug, the table provides DrugBank ID, drug name, approval status, category, number of drug-drug interactions, number of targets, number of associated genes, and indication information. By manually checking them, users can select the candidate drug(s) for further analysis.

After users determine the candidate drug(s), the DTome tool provides several data extraction options. For each data extraction option, the tool provides a single-system interface to output the corresponding summary and a results table, i.e., "Get DDI" for drug-drug interactions (Figure 3C), "Get Target" for drug-target interactions (Figure 3D), and "Get Related" for drug-gene associations (Figure 3E). Note that target-/gene-protein interactions are obtained using the "Get PPI" option from the output page of drug-target interactions or drug-associated genes (Figure 3F). For example, besides the downloadable drug-drug interaction table, the output page of "Get DDI" provides the number of drug-drug interactions, the number of drugs matched the users' requirement, and the number of the drugs having interactions with required drugs. These summaries and detailed interactions are useful for users to further examine the relationship between candidate drugs and relevant molecules and choose the interactions for further network construction. From the "Get Network" option, the users can select the interactions that they are interested in and then obtain a DTome network (Figure 3G).

### Application

To demonstrate the usefulness of the DTome tool, we constructed a DTome network for clozapine as an example case. The procedure for a list of candidate drugs is similar to that for an individual drug.

**Figure 4:**
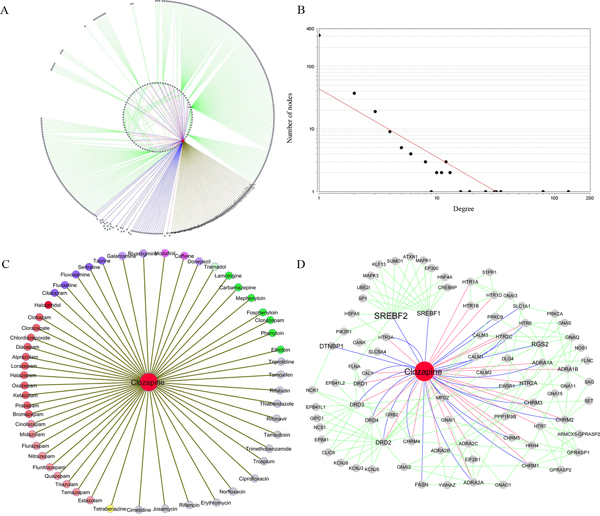
**Clozapine-target interactome network and its network characteristics**. **A) **Graphical representation of the clozapine-target interactome network. **B) **Degree distribution of all nodes (drugs, targets, genes, and proteins) in the clozapine-target interactome. The Y-axis represents the number of nodes with a specific degree. **C) **Graphical representation of clozapine adverse interaction drugs. According to Anatomical Therapeutic Chemical (ATC) classification systems, the nodes in different colors represent drugs belonging to the "nervous system" at the fourth level: N02A (light green), N03A (green), N05A (dark red), N05B (red), N05C (light red), N06A (purple), N06B (light purple), N06D (dark purple), and N07X (yellow). Nodes in grey with brackets represent drugs related to the "antiinfective for systemic use". Other nodes in grey represent drugs belonging to other categories with exception of above two categories. **D) **Graphical representation of clozapine-target interactions after removing the nodes with degree 1 and other drug nodes. An edge in red represents the relationship between clozapine and a target, an edge in blue represents the relationship between clozapine and an associated gene, and an edge in green represents the interaction between a target/gene and a protein from protein-protein interaction (PPI) data.

Clozapine, an atypical antipsychotic drug, is used to treat the symptoms of schizophrenia patient who do not respond to other medications [42,43]. After searching the database using "clozapine" in the "Drug Name" option, a summary table and several data extraction options mentioned previously appeared in the search output page. The summary table showed that clozapine had 54 drug-drug interactions, 26 primary targets, and 51 associated genes. After checking the select box following the drug name, the tool extracted all relationships for clozapine. By clicking "Get Network" option and selecting all data sources, we obtained a clozapine-target network. The network included 517 edges and 406 unique nodes. Among these nodes, 55 were drugs including clozapine and 54 other drugs having adverse interactions with clozapine, 26 were primary targets, 51 were associated genes and 292 were proteins with direct interactions with targets or genes (Figure 4A). There were 16 genes that existed in both primary targets and associated genes; they were *ADRA1A, ADRA2A, CHRM1, CHRM2, CHRM3, CHRM4, CHRM5, DRD1, DRD2, DRD3, DRD4, HRH1, HTR2A, HTR2C, HTR3A*, and *HTR6*.

Next, we noticed that the degree distribution of all nodes was strongly right-skewed as shown in Figure 4B, generated by NetworkAnalyzer tool, a Cytoscape network analysis plugin [44]. Thus, most nodes in this network had low degree while only a few nodes had higher connections, such as *DRD2, DTNBP1, HTR2A, RGS2, SREBF1*, and *SREBF2*.

To examine the classification of drugs that had adverse interactions with clozapine, we grouped them based on ATC classification system. Clozapine is an antipsychotic drug (N05A).Among the 54 drugs, 41 (75.93%) belonge to the category "Nervous system" and 6 (11.11%) belong to "Antiinfective for systemic use" (Figure 4C). Among the 41 drugs, 11 belong to anxiolytic drug (N05B), 9 belong to hypnotic and sedative drugs (N05C), 7 belong to antiepileptic drugs (N03A), and 5 belong to antidepressants (N06A).

**Figure 5:**
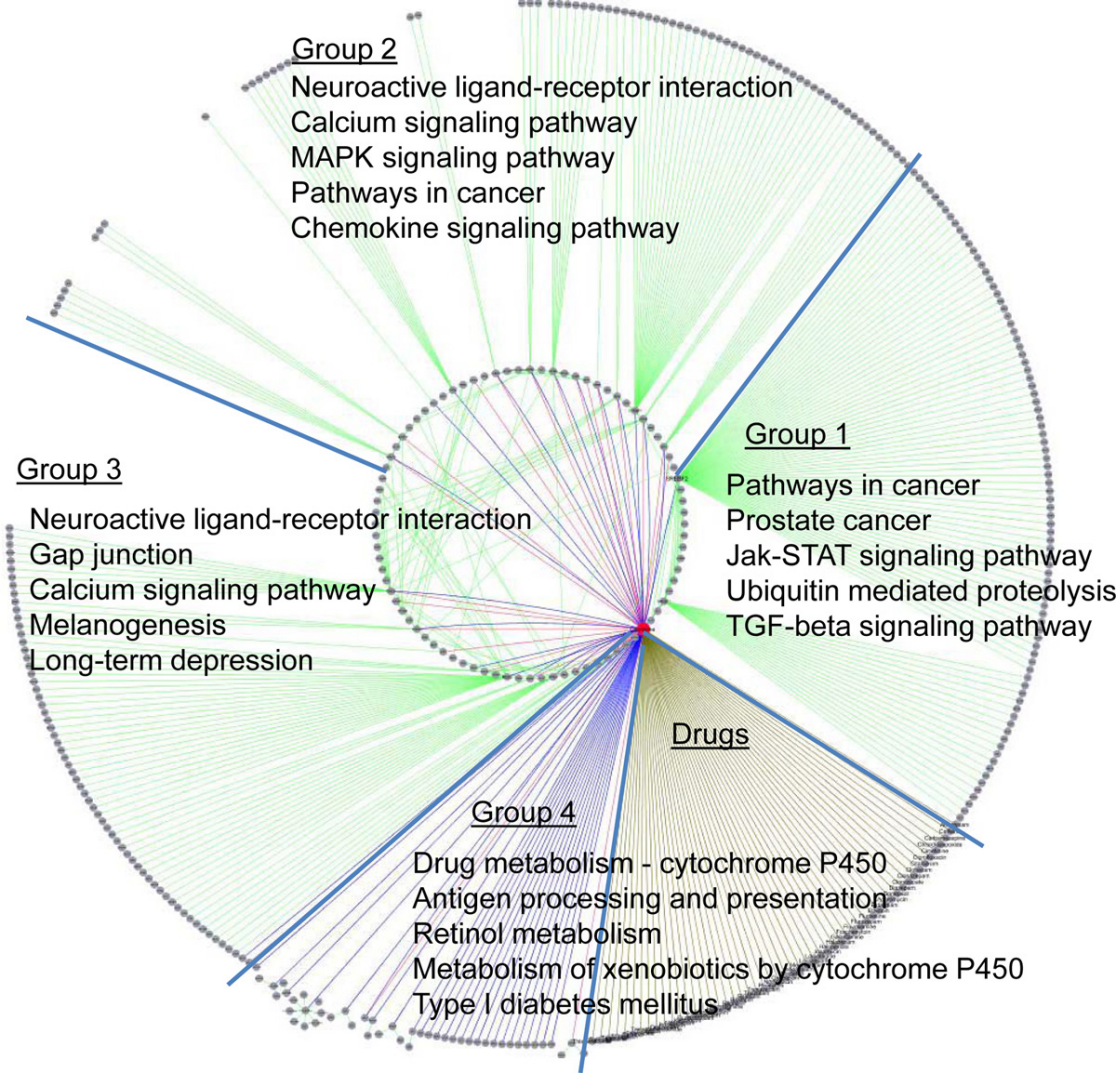
**Functional analysis of proteins in clozapine-target interactome network**. Based on the topological features of the clozapine-target interactome network, the proteins involved in the network could be generally classified into 4 groups. For each group, the top 5 enriched KEGG pathways were listed.

To further examine the interactions among targets, associated genes and other proteins from PPIs, we removed the drug nodes with the exception of clozapine node and the nodes with only one link. Overall, the simplified network formed three clusters, as shown in the Figure 4D. According to the clustering visualization in Figure 4A, five clusters are distinct to each other (i.e., four protein clusters and one drug cluster). To assess functional features of these groups, we performed the KEGG pathway enrichment analysis for four protein clusters. All groups showed high functional homogeneity with a Benjamini-Hochberg adjusted *P*-value < 0.01. The top 5 enriched KEGG pathways for each group were labelled in Figure 5. The 98 genes in group 1 mainly corresponded to the significant pathways associated with cancer and signalling pathways. Among the genes, only two genes, *SREBF1 *and *SREBF2*, were clozapine-associated genes. They encode sterol regulatory element binding transcription factors (TFs), which are reportedly associated with schizophrenia [45]. The 106 genes in group 2 were enriched in the "Neuroactive ligand-receptor interaction" and some signalling pathways. The 101 genes in group 3 were mainly associated with neurodevelopment-related pathways and some of the relative pathways. Group 2 and group 3 included most of primary targets and clozapine-associated genes. The 51 genes in group 4 were mainly linked to metabolism-related pathways. Therefore, according to the functional analysis, the proteins could be further categorized into three classes: transcription-related proteins, drug-related target/gene proteins, and metabolism-related proteins. Overall, these classes reflect the three main molecular layers in drug actions.

## Discussion

In this study, we have developed a web-based tool to search and integrate drug-target information to generate a DTome network for the candidate drug(s). As demonstrated by the construction of clozapine-target network and the follow-up network analyses, this tool is computationally efficient and represents a promising strategy to investigate the molecular mechanisms of drug actions. Therefore, this tool is unique and will be useful in the pharmacogenetics and pharmacogenomics areas.

This study mainly utilized two major drug datasets: DrugBank and PharmGKB and the integrative PPI data set from the PINA database. Thus, when interpreting these results from the datasets, one should keep in mind that the current workflow has its own limitations, including both drug data and human PPI data that are incomplete and are not error-free. Since several target-centered databases are available, such as Matador and SuperTarget [46], and the Therapeutic Target Database (TTD) [47], we will integrate more drug target datasets into the system to ameliorate the effects of data limitation in the future.

The network-based approach is emerging as a highly promising method to studying massive amount of omics data, and it has been successfully applied to numerous human disease studies [48,49]. In this study, we implemented the network pharmacy concept in a robust system by including the direct interactors from the PPI data into the drug-target network. This method is simple yet effective to obtain the relationship between the drug targets or drug-associated genes and their interacting proteins. Analyses of the DTome network for a specific drug or a list of drugs may allow for the identification of new drug targets and a better understanding of the molecular mechanisms of drug actions.

## Conclusions

In this study, we presented a computational workflow to generate a DTome network for a given drug or a list of drugs, and implemented the workflow through an online drug information search and integration tool. The tool is computationally efficient in generating and integrating drug-drug, drug-target, drug-associated, and target-protein interactions to build a DTome network. Our demonstration using the antipsychotic drug clozapine shows that the output of our system provides a starting point to further investigate the molecular mechanisms of drug actions, thereby suggesting its usefulness in the pharmacogenetics and pharmacogenomics research.

## Competing interests

The authors declare that they have no competing interests.

## Authors' contributions

JS prepared the data, participated in the development of the methods, database and web interface, carried out the data analysis, and contributed to the writing of the manuscript. YW participated in the database design and web interface development and contributed to the writing of the manuscript. HX provided advice on study design and tool development and contributed to the writing of the manuscript. ZZ participated in the method development and data analysis and contributed to the writing of the manuscript. All authors read and approved the final manuscript.
